# Skin-antigen specific antibodies are detected in UV irradiation and TLR7 agonist induced lupus-like disease in autoimmune prone NOD mice and in pediatric SLE

**DOI:** 10.1186/ar3977

**Published:** 2012-09-27

**Authors:** M Ghoreishi, L Tucker, JP Dutz

**Affiliations:** 1 University of British Columbia, Vancouver, BC, Canada

## Introduction

The role of environmental precipitants in autoimmunity such as systemic lupus erythematosus (SLE) remains unclear. We wished to determine whether UV alone or UV in the presence of TLR7 activation would induce lupus-like disease in an autoimmune mouse model, to explore underlying mechanism(s), and relevance in humans.

## Methods

Six-week-old female nonobese diabetic (NOD) mice received repeated weekly 5,000 j/m^2 ^UVB radiation or 25 μg topical imiquimod or both. Control mice were left untreated. For comparison, nonobese diabetic resistant (NOR) mice were treated with combination therapy (imiquimod + UV). Serum was collected for detection of anti-nuclear antibodies (ANA), desmoglein 3 (Dsg3) antibodies and IFNα by ELISA and detection of proinflammatory cytokines by cytokine bead array. Peripheral blood was collected for cell surface or intracellular staining using flow cytometry. PAS staining of kidney identified the presence of glomerulosclerosis. Dsg3 antibodies were also measured in the serum of children with SLE, type 1 diabetes (T1D) and normal controls.

## Results

Imiquimod treatment enhanced ANA and Dsg3 antibody production in NOD mice. Imiquimod + UV induced glomerulosclerosis. Systemic immune activation was detected following combination therapy but not single therapy as evidenced by IL-6, TNFα, IFNγ, and MCP-1. Serum IFNα was significantly elevated in NOD mice following combination therapy. Combination therapy upregulated TLR7 and IFNα expression in the peripheral blood PDCs of NOD but not NOR mice. Anti-Dsg3 antibodies were detected more frequently in children with SLE (5/19) than in children with T1D (1/9) or controls (0/10).

## Conclusion

These studies demonstrate that UV light combined with TLR7 engagement induces SLE-like disease in autoimmune prone animals. The presence of anti-Dsg3 antibodies in pediatric SLE suggests that skin-specific autoimmunity occurs in a subset of patients with SLE.

